# The UN Decade of healthy ageing in practice: A qualitative case study on perspectives of healthy ageing among older adults, healthcare professionals, and decision-makers in Norway

**DOI:** 10.1186/s12913-026-15363-8

**Published:** 2026-08-14

**Authors:** Mariya Bikova, Maria Nordheim Alme, Djenana Jalovcic, Hanne Tuntland

**Affiliations:** Department of Health and Functioning, Faculty of Health and Social Sciences, Western Norway University of Applied Sciences, Inndalsveien 28, Post Box 7030, Bergen, 5020 Norway

**Keywords:** Older adults, Long-term care, Age-friendly society, Ageism, Digital literacy

## Abstract

**Background:**

Global populations are ageing, making healthy ageing an increasingly important priority. In Norway, the number of people aged 70 and above is expected to double by 2050. The UN Decade of Healthy Ageing (2021–2030) guides Norway’s national programs on healthy ageing through its four action areas: combating ageism, age-friendly environments, integrated care and long-term care. The aim of this study is to explore how healthy ageing is understood and interpreted in Norway in light of the four action areas of UN’s Decade of Healthy Ageing and the enabler listening to diverse voices and meaningful engagement.

**Methods:**

The study draws on 23 semi-structured interviews with decision-makers, healthcare professionals, and older adults in a large urban Norwegian municipality. Participants were recruited through direct contact and through the municipality. Data from the interviews was transcribed verbatim and analysed collaboratively by the research team, following the principles of collective qualitative analysis.

**Results:**

Findings focus on three main themes: strategies for achieving mastery, mixed perceptions of ageing, and health services. Results indicate that while preparing for old age is seen as a collective responsibility, older adults are increasingly expected to prepare for old age. The emphasis on self-reliance risks marginalising vulnerable groups who lack necessary resources or support networks. The rapid digitalisation of everyday life is identified as significant barrier to equitable participation, underscoring the urgent need to foster more inclusive forms of active engagement. Municipal healthcare services are generally perceived as high-quality, but face resource challenges that create uncertainty among older adults.

**Conclusions:**

Healthy ageing in Norway is increasingly framed around participation and shared responsibility for ageing in place, reflecting the UN Decade of Healthy Ageing. However, growing individualisation and rapid digitalisation may reinforce inequities. Addressing this requires digital inclusion, intergenerational approaches, accessible environments, and sustained public investment, alongside efforts to combat ageism and promote more inclusive, collaborative forms of participation.

**Trial registration:**

Not applicable.

**Supplementary Information:**

The online version contains supplementary material available at 10.1186/s12913-026-15363-8.

## Introduction

Global population is ageing, with the number of older people projected to increase from 1.1 billion in 2023 to 1.4 billion by 2030 [1]. As populations age, enhancing quality of life in later years has become a global priority. In 2002 the World Health Organization (WHO) launched the Active Ageing Policy Framework, highlighting the importance for preparing for old age, self-care, age-friendly environments and intergenerational solidarity [2]. Healthy ageing has since become a much-used concept in theory, policy and practice. Although not with one clear definition, the concept refers to a lifelong process dependent on intrinsic factors, functional abilities and the interaction with the environment [3, 4]. In 2020, WHO launched the Decade of Healthy Ageing (2021–2030), aiming at improving the lives of older people, their families, and communities. Closely aligned with the Sustainable Development Goals (SDGs), the Decade of Healthy Ageing seeks to drive transformative changes in how societies perceive and support ageing. Four key action areas were defined for reaching this goal: combating ageism, age-friendly environments, integrated care and long-term care. All four action areas are strongly interconnected and perceived critical for obtaining healthy ageing [5].

Combating ageism involves addressing stereotypes, prejudices, and discrimination based on age, which is known to have a negative impact on the health and wellbeing of older adults [6]. Age-friendly environments are described as environments without physical and social barriers where policies, systems, services, products and technologies enable older adults to live dignified lives, irrespective of physical or mental capacities. Promoting age-friendly environments therefore involves several sectors, and includes arrangements for housing, transport, education, labour, information and communication, urban development, and health- and social services [7, 8]. Integrated care can be seen as a commitment to improve the quality and safety of care services, providing the assistance and care that is needed for good health and wellbeing [9]. Long-term care involves a range of services aiming to help people to live independently and safely when they cannot carry out routine and meaningful activities on their own. The services offered should be tailored to the specific needs of the older adults and can be provided at home, in the community or in various types of facilities [10]. The transformational change needed for reaching the goal of healthy ageing depends on strong collaborative actions. To support this, UN Decade has described four enablers: listening to diverse voices and enabling meaningful engagement, nurturing leadership and building capacity across sectors, connecting various stakeholders for collaboration and shared learning, and finally strengthening data, research and innovation [4].

### The Norwegian context

Norway is a social democratic welfare state with publicly financed healthcare services [11, 12]. Norwegian residents are legally entitled to healthcare services [13]. Municipalities in Norway are responsible for providing primary healthcare and social services to their residents. Services at the primary care level include access to general practitioner, rehabilitation, home care and long-term care services, and form a continuum of care tailored to individual needs. Home care includes home nursing and practical assistance, supported by telehealth and assistive technology to enable people live independently in their own homes for as long as possible [14]. Rehabilitation consists of interventions designed to optimise functioning and reduce disability in individuals with health conditions [15]. Reablement is a form of rehabilitation at home offered in 2/3 of the municipalities in Norway [16]. For older adults with moderate to severe physical and cognitive decline, municipalities provide nursing homes with short and long-term stays. Although largely subsidised by the state, there is a small fee for primary care services [14].

The demographic shift, with more older adults living with chronic diseases, puts pressure on both primary care and specialist health care, creating a growing concern about the sustainability of the welfare state. To address this challenge, national strategies and reforms, aligned with the UN Decade, have been adopted in Norway. The initiatives aim at shifting societal focus on ageing from burden to resource and creating age-friendly societies [17]; enhancing social engagement of older people [18]; facilitating ageing in place and postponing the need for institutional care [19]; optimising municipal healthcare service provision [20]. Common for all these governmental initiatives is the focus on creating opportunities for ageing in place by re-organising and optimising service provision, and by redistributing roles and responsibilities in a way that relies more on informal care, assistive technology and on service users’ individual responsibility. Hence, ageing in place focuses on supporting older adults to live at home for as long as possible [21]. This represents a shift in thinking where individuals are ascribed larger responsibility for planning for their old age as a way of reducing the burden on a pressured health- and social care system.

A Norwegian study explored how political documents convey assumptions about older adults, and how these shape practices and individual behaviours [22]. Studies have also explored how ageing-in-place initiatives are experienced by service users, family caregivers and municipal employees [23], how older adults experience active ageing in supported housing [24], and how home-dwelling older adults in smaller municipalities perceive their everyday lives [25]. However, research on how healthy ageing is understood by different interest-holders within municipal health services remains limited.

This study constitutes one of the cases within a broader multi-case study on Global Perspectives of Healthy Ageing conducted at the Western Norway University of Applied Sciences. Within this framework, the Norwegian study aims to explore how healthy ageing is understood and interpreted in Norway in light of the four action areas of UN’s Decade of Healthy Ageing and the enabler listening to diverse voices and meaningful engagement by answering the following research question: How are the four action areas and the enabler voice and meaningful engagement interpreted by older adults, healthcare professionals and decision-makers in a large urban Norwegian municipality?

## Methods

### Design and recruitment

This qualitative case study is based on individual interviews with older adults, healthcare professionals, and decision-makers in a larger urban municipality in Norway. A qualitative design was found appropriate given the study’s focus on understanding the perspectives of the three groups of informants. The semi-structured interview guides, developed for the multi-case study, cover the four action areas (see Appendix 1 and 2). The enabler *listening to diverse voices and enabling meaningful engagement* is chosen because it is directly relevant to the level of practice our study addresses and falls within the scope of municipal interest-holders’ influence. Informants were selected based on belonging to one of the three target groups, their relevant experiences, willingness to participate in the study and being able to provide informed consent. Approval to conduct the study was obtained from the municipality, and recruitment of home-dwelling older adults was done by municipal employees. The healthcare professionals were recruited through the municipality and by using the researchers’ own professional networks. The decision-makers were selected by the researchers following the principles of purposive sampling.

### Data collection

Twenty-three face-to-face semi-structured interviews were conducted with older adults, healthcare professionals, and decision-makers. The interviews were conducted in Norwegian language between February 2024 and August 2024. The duration of the interviews was between 26 min and 1 h and 15 min. The interviews with older adults were conducted in their preferred setting, ensuring a comfortable and safe environment to facilitate open conversations. Options included their homes or other suitable locations, allowing flexibility based on individual needs. For decision-makers and healthcare professionals, interviews were carried out at their workplaces, either physically or through digital platforms. All interviews were audio-recorded and fully transcribed using the software Autotekst through safe login.

The study included older adults, seven women and three men, aged 68 to 87 years, all of whom lived independently in their own homes at the time of the interview. The eight healthcare professionals were aged 30 to 73 years and included six women and two men. Their professional backgrounds encompassed physiotherapy, occupational therapy, geriatric nursing, medicine, and geriatric medicine; one participant was retired at the time of the interview. The decision-maker group consisted of five individuals aged 30 to 63 years, including three women and two men. The decision-makers had managerial positions at various administrative levels or served as elected representatives in local government. The size of the informant group was decided by a pragmatic balance between the availability of the participants with specific expertise and the scope of the study.

### Data analysis

This study employed collective qualitative analysis, a methodological approach emphasising group-based interpretation and collaborative synthesis of qualitative data [26]. Drawing inspiration from Braun and Clarke’s thematic analysis framework [27], the approach was operationalized through Eggebø’s four-step model: (1) reviewing the data, (2) mapping (3) sorting, and (4) developing an outline and workplan for the manuscript. The analytical process began with summarising interview transcripts and highlighting key themes, contextual nuances, and notable emotional expressions or interactional dynamics observed during the interviews. Researchers produced a one-page summary per interview, incorporating anonymised background information and salient insights. This preparatory work ensured that team members were thoroughly familiar with the data prior to a collaborative workshop, that served as a core of the collective analysis process. During this workshop, researchers systematically reviewed the data, conducted thematic mapping, and organised the data, leading to the identification of sub-themes. The process resulted in a formulation of a focused research question and a structured outline for the study. Following the workshop, the team engaged in an iterative process to further refine and develop the themes presented in this paper. To illustrate patterns of shared meaning in the developed themes, quotes are inserted.

The multidisciplinary research team consist of four female researchers with Norwegian and international backgrounds aged 46–64 years. All researchers have experience in health and ageing-related topics from different perspectives (sociology, occupational therapy, rehabilitation, physiology). The aim has been to understand the data from different perspectives, while ensuring that the voices of the participants remain central.

### Use of software and artificial intelligence

The software Autotekst was used for transcription and translation of the interviews. The AI software NotebookLM was used to support the development of summaries, and the AI-software M365 Copilot for condensing and improving the flow of text. All use of AI has been thoroughly evaluated by the researchers in line with the recommendations in Levitt [28].

## Results

### Themes

Three main themes were developed from the analysis of the empirical material: *strategies for achieving mastery*,* mixed perceptions of ageing*, and *health services.* These themes shed light on how older adults understand their ability to manage and control their lives. They also communicate important aspects of how decision-makers and healthcare professionals perceive and facilitate healthy ageing. The themes are not mutually exclusive. Rather, they intervene and inform each other, adding depth and complexity to the analysis. Table 1 gives an overview of the themes and sub-themes. 

**Table 1:** Understanding healthy ageing in Norway: Thematic analysis of interest-holders’ perspectives

Main theme	Sub-theme	Dimensions of the sub-theme
Strategies for achieving mastery	Planning for old age	Navigating digitalisation Accessible housing Inclusive urban planning
	Enabling meaningful participation	Importance of individual resources Paid work, organised activities and voluntary work Barriers to participation
Mixed perceptions of ageing	Societal narratives on ageing	The silver tsunami Popular culture leading to discrimination Reframing ageing and older people Role models
	Older adults’ self-perceptions	Feeling respected Self-ageism Goal of independence
Health services	Experiences with municipal care	Service aim, quality and duration Ambivalence towards long-term care Long waiting lists
	Discrepancy between needs and possibilities	Political aim for involvement from family, community and voluntary sector Intersectoral collaboration for better service design

### Strategies for achieving mastery

The theme *strategies for achieving mastery* is related to how the different groups in our study prepare for old age, or facilitate preparation of others through their professional practice. This theme also explores conditions for meaningful social participation.

#### Planning for old age

The empirical material shows what planning for old age entails and how older adults prepare for the many factors shaping their everyday lives. It also reflects broader societal factors beyond their control that still affect their ability to manage and shape their lives.

Several public services, including services related to healthcare, are increasingly offered digitally and older adults in the study recognise the importance of building digital competences as a way of managing their everyday lives.

Most important is probably to keep up with the digital so that you can use the services. I wanted to call a doctor and book an appointment. However, I visited Health Norway’s website and did it there. It wasn’t that difficult. It’s important to be part of digital development [otherwise] you are dependent on others (Older adult 1).

A lack of digital competence can seriously compromise personal autonomy, hinder access to essential information, and limit social participation. Healthcare professionals in our study noted that older adults frequently request help navigating vital digital services like banking and healthcare, often facing isolation when unable to access online details about community activities.

Regarding the access to services, I find it problematic that we are very much in a time of digitalisation. We are reducing autonomy. The more digitalised the everyday life, the less autonomous the average older person becomes (Healthcare professional 4).

Moreover, informants describe that the need for assistance can be greater among older adults with a foreign background and those without family members to help them navigate the digital sphere. Digital competence is viewed as a shared responsibility between individuals and society.

Planning for later life, particularly securing age-friendly housing, is seen as key to supporting future independence and social connections, and as a shared responsibility between individuals and the public sector. Decision-makers emphasise this by stating, *“We all have responsibility for planning for old age by ensuring we can live in age-friendly housing*” (*Municipal decision-maker 2*), attributing this to changing societal conditions, particularly older adults’ improved financial situation compared to previous generations. This is illustrated by an older adult, who describes how moving to a new home increased social interaction with peers and enhanced well-being.

Mostly older people live here. No children. It’s a bit sad, but there are very nice people living here. You often strike up a conversation when we’re out for a walk. Everyone says hello. You get in a very good mood by going out for a walk (Older adult 4).

While the initiatives described above are mainly individual preparations for old age, adapting the physical environment to meet the needs of an ageing population requires planning at the societal level. The decision-makers underscore the importance of universal design and accessible infrastructure in enabling mobility and participation. They also highlight the intergenerational living and social integration, suggesting housing models that foster interaction between age groups to enhance mutual support and reduce loneliness.

Children and young people can benefit from living closer to older people. Regardless of functional ability, they can give each other a lot. Security, support, knowledge, practical help, physical, social and psychological care (Municipal decision-maker 1).

#### Meaningful participation

Access to resources such as financial means, network of former colleagues, family and friends, is essential for staying socially active. Many of the older adults in our study report having sufficient financial resources to engage in social and cultural activities, and to cover healthcare costs. Healthcare professionals share the view that most older adults in Norway have sufficient financial resources to live normal lives, even joking that “*materially and financially*,* we’ve hit the jackpot by being born here*” (*Healthcare professional 2*). However, they also recognise that some older adults do worry about finances.

Social networks are another important resource highlighted by participants. Those with employment histories often maintain networks of colleagues and friends that provide information, social contact, and opportunities for small jobs after retirement. Older adults with such paid work find it meaningful and enjoyable in daily life.

I work part-time in a 30% teaching position, and the children were so happy to see me again. Being part of a collegium, using my experience, and feeling that I make a difference fill me with joy (Older adult 8).

Beyond their volunteer work, many of the study’s older adults engage in organised physical activity, including group training, swimming, and hiking in the mountains.

Although most older adults in our study engage in activities outside the home, the Norwegian climate and culture can pose barriers to participation for some. Cold weather and limited daylight during winter may reduce both motivation and opportunities to go out. In addition, Norwegians are often seen as quite private and may rarely initiate spontaneous visits to neighbours or family, which can limit social networks in later life. One participant noted that he and his wife have little contact with their neighbours; although they would like more interaction, they do not take the initiative themselves.

We do have a little bit of contact with those on our floor. But it’s not that they invite us to their apartment. I miss that. But I am a bit passive myself (Older adult 3).

As shown above, participation depends on individuals’ willingness and capacity to engage in social and community life, but also on broader societal structures, including inclusive policies and political attention to age-related issues. Decision-makers identified structural barriers such as workplace norms and pension regulations that may limit continued participation. Greater flexibility in work arrangements and policy reforms were suggested as key to better utilising the resources of older adults.

Participation also involves advocating for older adults’ interests. However, there is a discrepancy between decision-makers’ views and the experiences of older adults and healthcare professionals. Decision-makers claim older adults’ voices are represented through bodies such as the Senior Citizens’ Council and are “certainly being heard” (Municipal decision-maker 2). In contrast, many older adults choose not to engage in political processes, often due to feeling “too old” or not competent enough. Healthcare professionals also recognise that older adults’ interests are not always adequately represented.

Older people are not the strongest lobbying group, and they are fully aware of that. There is a need for more passionate advocates who have the energy to work with politicians and make a difference. Once you leave the workforce, you lose a key platform to make an impact (Healthcare professional 1).

### Mixed perceptions of ageing

The theme *mixed perceptions of ageing* reflects both societal views of older adults and how older adults see themselves. Examining these perspectives helps to better understand how they shape healthy ageing.

#### Societal narratives on ageing

Reflecting on ageing, decision-makers and healthcare professionals refer to the ‘silver tsunami’ and its challenges, but also to the untapped potential of older adults. Decision-makers particularly highlight the growing gap between care needs and service availability, while also discussing solutions such as age-friendly environments and enabling more people to age in place.

We are expected to focus on age-friendly cities and ageing in place, though it’s unclear whether older adults are seen as a burden or a resource. We are preparing for a future with more older people needing care and fewer caregivers. Welfare technology can be part of the solution, but can’t replace a person (Municipal decision-maker 1).

Healthcare professionals and decision-makers recognise that portraying older adults as a financial burden overlooks their substantial contributions to families, communities and society. They also note the influence of media and popular culture on these perceptions, and highlight the need for updated role models “[…] *someone to follow and to identify with”* (*Healthcare professional 5*), as well as greater awareness of how we talk about older people.

The ‘silver tsunami’ is viewed as problematic. If we as a society are to manage the demographic shift, we have to view it as a resource. It is indeed a huge resource. Many resourceful people are now seniors (Municipal decision-maker 2).

#### Older persons’ self-perceptions

The way older people view themselves and others influences their well-being and motivation for participation. In our study, older adults report feeling respected by their families and society, describing themselves as active and engaged, and as planning ahead for old age. However, when asked how they advocate for their own needs and interests, only a few report using existing channels.

We have a public bus which they were about to stop running, so we decided to take action. I contacted local politicians […] and went to the local newspaper. The bus is very important for us older people. After that, they did not dare touch it anymore (Older adult 1).

Older adults in the study report not feeling eloquent enough to participate in public debates. They also feel that rapid societal changes are hard to keep up with, which reduces motivation. Some doubt that individual engagement will lead to change. While decision-makers call for greater involvement, they also acknowledge the challenges older people face in voicing their needs.

It’s difficult to be an older person and know you represent the whole group. We have a strong focus on facilitating participation, but they need the competence to do so effectively. They must understand they share responsibility for decisions and have a sense of collective ownership (Municipal decision-maker 4).

According to one municipal decision-maker, a relative lack of engagement regarding the conditions of aging may simply be characteristic of ‘this specific generation’ of older adults.

Older people are perhaps less likely to reach out and express dissatisfaction. They settle with what they get and do not want to be ungrateful. But then, they are dependent on resourceful relatives who can speak up for them (Municipal decision-maker 3).

These findings reflect older adults’ internalised perceptions of their societal role. As one participant noted, *“We*,* older people*,* tend not to take the place we should in society. But it’s really up to us”* (Older adult 1). This internal dynamic is complex, as another informant observed that older adults may also reproduce these biases and discriminate against one another, *“I see discrimination in the way older people treat each other. Those who are not entirely comfortable with digital tools are bullying those of us who use them”* (Older adult 7).

Healthcare professionals also observe that older adults can engage in self-discrimination driven by these negative self-perceptions. As one practitioner noted,

There is discrimination in all societies, but there is also self-discrimination. As a practitioner, I have observed some people feel that once they reach 60, 62 or 65, it is better to withdraw and stay at home (Healthcare professional 3).

#### Health services

This theme explores older adults’ views and experiences of municipal healthcare services. It also examines how decision-makers and healthcare professionals respond to increasing service demands within the constraints of limited municipal budgets.

#### Experiences with municipal care

Many of the older adults in our study had used municipal healthcare services such as home care, reablement, or inpatient rehabilitation. Overall, they expressed satisfaction with these services, particularly with rehabilitation and reablement. Several participants noted that this tailored approach contributed to a faster recovery, *“Reablement changed everything. It helped me get quickly back on my feet”* (Older adult 7). Some were provided with assistive devices and welfare technology to promote ageing in place. Others report having to ‘fight’ for getting rehabilitation care or having to wait long time before the reablement team came to their homes and started the reablement process.

Views on long-term nursing home care were largely based on what older adults had heard or experienced during visits or short stays. Aware of long waiting lists, some expressed ambivalence toward the service. Others, despite modest expectations, were more accepting of the possibility of future admission and recognised that places in such facilities are scarce.

If I have to, I have to, but it’s not something I wish for. You hear so many things about nursing homes, and it makes you think twice. Still, places are limited, so if you are offered one, you have to accept it (Older adult 3).

Some older adults in our study expressed a preference for ageing in place rather than moving to a nursing home. At the same time, they acknowledged the challenges of living at home, particularly related to declining physical abilities and concerns about loneliness and isolation. As one participant explained,

Staying at home is a dilemma. I will be alone most of the day, with only brief visits for healthcare services. Apart from that, I will be completely alone. But I suppose I might feel just as alone in a nursing home (Older adult 5).

#### Discrepancy between needs and possibilities

In the context of tight municipal budgets and demographic pressure on the health sector, new distribution of tasks that implies more involvement of relatives and volunteers in non-clinical tasks, is seen as necessary. Decision-makers emphasise the importance of informal care, but at the same time, recognise that people’s support networks differ in regard to family and community involvement.

We know that municipalities will face tighter budgets and will have to handle more tasks. Therefore, we need to become much better at engaging the voluntary sector and tapping into the significant resource that family caregivers represent (Municipal decision-maker 3).

Healthcare professionals also recognise that, given the shortage of healthcare personnel, relatives and volunteers need to play a greater role in providing the social aspects of care.

If an older person expects healthcare staff to act as companions, they are mistaken. Healthcare staff are there to do their job. It is volunteers and family who help ensure quality of life. They attend to the social aspects of care and go the extra mile (Healthcare professional 8).

Both decision-makers and healthcare professionals acknowledge that certain groups of older adults, particularly those with cognitive decline, functional limitations, or a foreign background may face discrimination, as they often encounter difficulties in accessing information, applying for services, or expressing their needs

People with cognitive decline are more likely to be exposed to discrimination. They are often sidelined (Healthcare professional 8).

If not directly supported by close family members, such groups may be deprived of services simply because they are unable to apply. This also applies to older adults with other functional challenges. Healthcare professionals express concern that resource constraints in the municipal sector may be compromising the safety of vulnerable older adults, including those with hearing, visual, cognitive, or mental health challenges. Meeting the complex needs of a diverse ageing population amid budget cuts and shortages of healthcare personnel remains a significant challenge.

I think that the public health services are now a bit on the back foot, because needs are emerging that they are not equipped for. There are long waiting lists. Many older adults probably experience great frustration. Many have complex health problems that can’t be solved by one type of service only (Healthcare professional 5).

Decision-makers point out that in this situation, it is necessary to adjust public expectations regarding what health services can realistically deliver, while simultaneously finding new pathways for cross-sectoral collaboration. This shift is driven by stark resource limits, as one official noted, “*we do not even have enough healthcare staff to perform basic tasks*” (Municipal decision-maker 4).

## Discussion

In the following, we discuss our findings in light of the Decade’s four action areas, and the enabler voice and meaningful participation.

### Combating ageism

This action area focuses on changing how ageing is understood and experienced. In our material, the main theme *mixed perceptions of ageing* shed light on how society thinks, feels and acts towards older people and how older people perceive themselves. Our data reveal mixed perceptions at both societal and individual level. Negative narratives of older people as being ‘silver tsunami’ and financial burden co-exist with perceptions of older people as an important resource to their families, communities and society. Media and popular culture influence ageist attitudes, underscoring the need for new role models and greater awareness of how we speak about older adults.

At the individual level, participants describe themselves as active, resourceful and independent. Recent studies of community- and home-dwelling older adults in Norway show that many maintain positive self-perceptions despite physical changes and losses, and often wish to set a good example for younger generations [25, 29]. Some of the older adults in our study were engaged in advocacy and community issues, while others actively chose not to. This suggests that older adults practice both self-ageism and ageism towards their peers. These internalised and explicit biases emphasise the urgent need for new role models, revised societal narratives, and environments that actively foster older adults’ capabilities.

### Age-friendly environments

Developing age-friendly environments that support dignified living, regardless of functional ability, is the UN’s second action area. In our study, this is reflected in the theme *strategies for achieving mastery*, highlighting preparation at both societal and individual levels. At the societal level, this includes inclusive urban planning, flexible pension systems, and accessible information and services. At the individual level, it involves moving to suitable housing, maintaining social connections, keeping up with digital developments, and engaging in community life. Our findings align with research showing that organised activities and voluntary work are key to sustaining social connectedness and meaning in later life [25]. Research on home-dwelling older adults similarly shows active preparations for ageing, such as adapting homes, relocating to age-friendly housing, staying physically active, and maintaining social ties [30]. For those living in supported housing, participation in organised activities is also central to experiencing active and healthy ageing [24].

A critical, yet challenging, aspect of preparing for old age is keeping pace with digital developments. All three informant groups expressed concern that the rapid digitalisation of everyday life, including the shift to digital information, increases vulnerability among already marginalised groups, such as older adults with foreign backgrounds or physical challenges. Recent studies support this, showing that many older adults experience modern technology as a barrier and even a threat to participation [25]. Related research suggests that digital competence should be considered part of older adults’ independence literacy, requiring responsibility from both interest-holders and older adults themselves [30].

### Integrated care and long-term care

For healthcare professionals, a key component of an age-friendly society is the provision of high-quality, integrated, and responsive long-term care. This aligns with the UN’s third and fourth action areas and is reflected in the theme *health services*. Our findings show that delivering timely, accessible, and high-quality care remains challenging due to tight municipal budgets and shortages of healthcare personnel. While older adults are generally satisfied with the quality of municipal care, particularly reablement services, they also report long waiting times for accessing these services.

Comparable studies show that home-dwelling older adults in Norway are often sceptical of municipal services due to perceived poor quality and staff shortages [25, 30]. Research on ageing in place across six Norwegian municipalities found that services frequently fail to meet the needs of frail older adults because of limited competence and insufficient time for medical nursing tasks [30]. Concerns have also been raised about the decline of the more personal aspects of care, the so-called social care such as spending time talking with older adults due to time constraints [25, 31]. This is supported by studies showing that municipal healthcare workers often deprioritise social care because of limited time and staffing [32].

Healthcare professionals and decision-makers in our study call for greater involvement from families, volunteers, and the community to provide social care and alleviate pressure on municipal services. This aligns with research highlighting the essential role of informal caregivers in bridging service gaps to enable frail older adults to remain at home [30]. However, our findings show that older adults feel ambivalent about long-term institutional care due to concerns over availability, waiting lists, and quality, preferring to age in place only if adequate home care is provided. Similar ambivalence has been reported in studies from other Norwegian municipalities [25]. Furthermore, healthcare professionals in our study and in other municipalities express concern that limited nursing home capacity disproportionately affects vulnerable groups, particularly those with cognitive decline, and may force ageing in place even when it is no longer safe [30].

### Voice and meaningful engagement

The Decade emphasises meaningful engagement of older adults as a crucial *enabler* for improving their lives, recognising them as rights-holders and agents of change rather than as passive recipients of care. The aim is to ensure that policies regarding the conditions for ageing are inclusive by incorporating the diverse voices and experiences of the older population. Our study indicates that while older adults are actively engaged in community life, they show less interest in advocating for their own ageing needs even when they are aware of the existing channels.

Individual interests, as well as professional and educational backgrounds, influence engagement. In our study, older adults who advocated for their rights typically had prior advocacy experience. Self-perceptions also appear important. Many view themselves as “too old”, insufficiently articulate, or unable to keep up with rapid societal and digital developments. Such internalised ageist perceptions may hinder engagement. Moreover, many of the oldest old place a high value on modesty and may therefore refrain from complaining [33]. At the same time, decision-makers call for increased participation among older adults and promote this through formal public structures. However, it remains unclear whether these structures align with older adults’ preferred forms of engagement.

### Healthy ageing in Norway

Like many countries, Norway is reorganising its healthcare sector to counter demographic shifts and rising economic pressures. Within this context, healthy ageing is often conflated with ‘active ageing’, ‘successful ageing’, and ‘ageing in place’. While distinct, these concepts collectively shift the focus from passive caretaking to empowering older adults to remain independent and socially active. However, while this shift may ease the burden on municipal services, it simultaneously redefines the classic welfare state by quietly transferring systemic responsibility onto individuals, families, and the voluntary sector [22, 23, 34].

Older adults often take informed steps to prepare for old age, supported by public initiatives such as inclusive urban planning and flexible pensions. However, significant barriers to participation remain, particularly for those with foreign backgrounds, limited digital skills, or physical and cognitive impairments, potentially restricting access to essential healthcare services. Moreover, the capacity to support healthy ageing varies widely across Norway. Our findings, in line with previous research [30], show that factors such as municipal geography – urban or rural, large or small, and fiscal capacity directly shape the quality of service provision.

This study was conducted in a large urban Norwegian municipality characterised by a substantial population base and highly centralised services. These structural conditions may account for the relatively high levels of satisfaction expressed by participants, despite considerable waiting lists. In contrast, comparable studies show that smaller, rural municipalities where limited staffing and vast geographical distances pose significant challenges, face greater difficulties in delivering consistent, person-centered, and integrated care [25, 30]. Healthcare professionals express concern that long waiting lists may negatively affect older adults with cognitive decline and physical impairments. For these groups, ageing in place is not always synonymous with healthy ageing, particularly when municipal services lack the specialised resources and time needed to support them effectively.

### Strengths and limitations

Combining the perspectives of older adults, healthcare professionals, and decision-makers provides a broad understanding of factors that promote or hinder healthy ageing within the Norwegian welfare state. This multi-stakeholder approach fosters a deeper understanding of how contextual factors intersect at both the individual and societal levels to shape the conditions for healthy ageing. The inclusion of healthcare professionals from diverse backgrounds, including physiotherapists, occupational therapists, geriatric nurses, geriatricians, and general practitioners, presents both a strength and a limitation. On one hand, this variety introduces an interprofessional perspective to the analysis. On the other hand, balancing the breadth of these varied viewpoints may have compromised the analytical depth that a more homogeneous professional cohort could have provided. Similarly, the decision-maker sample spans a variety of managerial positions across different administrative levels, offering valuable insight into how policy mandates translate into local practice. The sample of older adults is diverse in terms of professional background but relatively homogeneous with regard to socioeconomic, ethnic, national, and geographic characteristics. As such, the findings reflect situated and contextual knowledge. Future research including more diverse participants and municipalities may help determine the transferability of these findings to other settings. Moreover, future research should explore how older adults can be supported through training that helps them recognise ageist assumptions and develop the skills needed to effectively advocate for themselves.

## Conclusion

The study indicates that understandings of healthy ageing in Norway are increasingly shaped by expectations of meaningful participation and shared responsibility for ageing in place, in line with the goals of the UN Decade of Healthy Ageing. A growing individualisation of responsibility may reduce equity in service provision, particularly for those with lower socioeconomic status, impairments, or migrant backgrounds, while rapid digitalisation can further limit access to services and participation. Addressing this requires stronger digital inclusion, intergenerational connections, and accessible environments, supported by sustained public investment. Combating ageism is also crucial, as it shapes both societal narratives and older adults’ self-perceptions. The gap between formal participation structures and lived experiences highlights the need for more inclusive, collaborative approaches that enable engagement on older adults’ own terms.

## Supplementary Information

Below is the link to the electronic supplementary material.

Supplementary Material 1

Supplementary Material 2

Supplementary Material 3

## Data Availability

The data underlying this study were used under license and are not publicly available. Data may be requested from the corresponding author.
