# Supplementary material for: The UN Decade of healthy ageing in practice: A qualitative case study on perspectives of healthy ageing among older adults, healthcare professionals, and decision-makers in Norway
Source: BMC Health Serv Res. 2026 Aug 14;26:1144. doi: 10.1186/s12913-026-15363-8 (PMC13488574; doi:10.1186/s12913-026-15363-8)
Supplement: Supplementary file 1 [file 12913_2026_15363_MOESM1_ESM.docx]

Appendix 1: Interview guide for older persons

Global Perspectives on Healthy Ageing (GPHA Study)

Age:_____________________________

Gender: _________________________

Having a partner: yes/no

Interviewer name: _________________

Study code: ______________________

**1.0 Preamble**

Healthy ageing is developing and maintaining the functional ability that enables well-being in older age. In this study, we are interested to know more about you and how you interact with your environment. We are also interested to know about your opinions, perspectives, and your experiences living in your community.

**2.0 Introduction section**

This section is about providing background information on the informant.

#1: Can you tell me a little about yourself? *Prompt: What is your age, do you live alone or with family, what are your daily activities?*

#2: Can you tell me the *year* you were born, and were you born here or somewhere else?

#3: What sort of work did you do before retiring? *Prompt: Do you currently work, or what kind of work did you do before?*

#4: What kinds of things or activities do you value? Prompt: What kind of activities or hobbies do you enjoy and where you find meaning.

**3.0 Age friendly environments section**

This section is about to what extent communities foster the abilities of older people.

#5: In this question, we would like you to describe the place where you live. How do you go about daily activities? What kind of transportation do you use to go shopping or visit friends? What do you think about the environment where you live?

#6: Where you live, what kinds of learning activities or ways to get involved in your community exist? Have you participated in learning or social activities in the last month or so? If yes, describe for me the most recent activity you participated in? *Prompts: What did you do? Who organised it and where?*

#7: Do you have access to adequate pensions and social assistance to live? *(Prompts: If no, how do you get the financial resources you need to live? Do you work? If yes, how do you find work? Describe any barriers to getting work or retaining it. Are there barriers to retaining or hiring work for you?*

#8: Do you have access caregivers to help if you needed them to? If you need help with daily activities, how do you get it? Who helps you? When you are sick, who looks after you?

#9 In your experience, are there opportunities for you to raise your voice and have a discussion on health and well-being for you? Have you participated in any organised discussions about health and well-being in your community? If yes, describe your experience. If no, would you like that opportunity?

**4.0 Combatting ageism section**

This section is about the common attitudes towards older people.

#10: Describe your role within your family? How would you describe your relationship with your family? How do you feel your family members treat you? How would you describe their view of older people? How are older people treated in your community? Can you give me an example?

\# 11 Describe for me how do other people act towards older people in your community? How does their behavior make you feel? Can you give me an example? What do people in your community think about ageing? How does that make you feel?

#12: Politicians and other decision makers generally create policies or rules. How do you think decision makers act or feel toward part of the population that are older? Can you give me an example?

#13: Do you know of any policies or rules that ensure that persons are treated fairly, equitably and with dignity as they age? *Prompt: If yes, please provide an example of a policy, and how it is used; if no, move on.*

**5.0 Integrated care section**

This section is about to what extent communities deliver person-centred integrated care and services that respond to older people`s needs.

#14: Can you tell me a little about your experiences and/or perceptions of the health care system? Think about the last time you used health care services. Can you tell me more about it? *Prompts: Was it easy to get services need? How pleased/satisfied were you with the care you received? Do they take your needs and wishes into consideration?*

#15: If you needed to get services from more than one provider, how did you think that your care was coordinated between providers? Describe your experience in accessing several providers.

#16 Is cost a barrier to getting the care you need?

**6.0 Long-term care section**

Older people may reach a point when they can no longer care for themselves without support and assistance. This section is about to what extent communities provide access to long-term care for older people. (Operational definition: “Long-term care services include traditional health service such as management of chronic geriatric conditions, rehabilitation, palliation, promotion and preventative services”).

#17a. *If “not in” long term care*: When you might need more help with your daily activities, do you think that you would be able to get it? What if you needed to go to a residential facility or older person's home? What do you think about older people's homes? What do your family/community members think about having a loved one in a home for older people?

*#*17b. *If “in” long term care setting*: Can you tell me a little about what it’s like to live here? Do the staff provide what you need? How are the social interactions and relationships in this setting?

**7.0 Conclusion section**

I want to thank you so much for taking the time to talk with me today. We would be happy to share the results of this study if you are interested.

#19 Are you interested in receiving the conclusions from this study (circle one)?

Yes	No

**END of INTERVIEW GUIDE**
