# Supplementary material for: The UN Decade of healthy ageing in practice: A qualitative case study on perspectives of healthy ageing among older adults, healthcare professionals, and decision-makers in Norway
Source: BMC Health Serv Res. 2026 Aug 14;26:1144. doi: 10.1186/s12913-026-15363-8 (PMC13488574; doi:10.1186/s12913-026-15363-8)
Supplement: Supplementary file 2 [file 12913_2026_15363_MOESM2_ESM.docx]

Appendix 2: Interview guide for healthcare professionals and decision-makers

**Global Perspectives on Healthy Ageing (GPHA Study)**

**Interview Guide: “Health Provider” and “Decision Maker Participant”**

Date: ___________________________

Interviewee code: ________________

Interviewee category: Formal health provider _______________

Policy or decision maker_______________

Interviewer name: _________________

**1.0 Preamble**

The UN Decade of Healthy Ageing is from 2021–2030. The Decade is “a global collaboration, aligned with the last ten years of the Sustainable Development Goals, that brings together governments, civil society, international agencies, professionals, academia, the media, and the private sector to improve the lives of older people, their families, and the communities in which they live”. The goal of the Decade is to optimise older persons` functional ability. This interview is about four action areas and one enabler presented in the UN Decade of healthy ageing report.

**2.0 Introduction section**

This section is about providing background information on the informant.

#1: Can you tell me a little about yourself (prompt: What do you do/ what is your profession and/or position, how long have you been doing this, etc...

#2: Can you tell me how old you are, and where you are from, here or somewhere else?

Part 1 – The four action areas

**3.0 Age friendly environments section**

This section is about to what extent communities foster the abilities of older people.

#3: What do you consider to be an age-friendly environment? *Prompt: Physical environment, social environment.*

#4: Do older people have access to adequate pensions and social assistance to live? *Prompt: What kind of? Why or why not?*

#5: What about possible income-generating activities/employment for older adults?

#6: From your perspective, what kind of access do older people have to things like lifelong learning activities? *Prompt: Do older people have access to education possibilities?*

**4.0 Combatting ageism section**

This section is about the common attitudes towards older people.

#7: What do you think are the opinions of the overall community towards older people? What might influence people here to have positive or negative perceptions about older people? Can you give me some examples of what you mean?

#8: Are there any groups that in particular are met with ageist attitudes in your country/region/district/community? *Prompt: Based on gender, ethnicity, functional ability/disability.*

#9: Politicians and other decision makers generally create policies or rules. In your experience, how do decision makers act or feel towards the older part of the population? Can you give me some examples?

**5.0 Integrated care section**

This section is about to what extent communities deliver person-centred integrated care and services that respond to older people`s needs.

#10: Essential health services include prevention, promotion, curative, rehabilitative, palliative and end-of life care. Medicines and vaccines, dental care and assistive technologies are also part of it. To what extent do these health services exist / or are accessible for older persons in your country/district/community?

#11: To what extent do health services respond to older persons` needs? Are health services accessible, affordable, good-quality, effective and safe? Can you give examples?

#12: Primary care is the main entry point for many older persons into the health system. To what extent does your country/district/community provide primary health services for older people? If low provision – what are the barriers? *Prompts: Competent workforce, appropriate legislation/regulation or sufficient sustainable funding.*

**6.0 Long-term care section**

Older people may reach a point in their lives when they can no longer care for themselves without support and assistance. This section is about to what extent communities provide access to long-term care for older people who need it. Long-term care is defined as “...services that include traditional health service such as management of chronic geriatric conditions, rehabilitation, palliation, promotion and preventative services.”)

#13 To what extent is formal long-term care available, and of what kind? *Prompt: Residential care, assisted living, day-care, respite care and home-care.* Is it provided by the government, not-for-profit or for-profit-organisations?

#14: To what extent does long-term care rely of informal care provided by family or others? Who are the informal caregivers? To what extent do older people have access to informal caregivers?

Part 2 – the enabler

**7.0 Voice and engagement section**

This section is about to what extent the informant`s country/region/community is listening to diverse voices and enabling meaningful engagement of older persons, family, caregivers, young people and communities.

#15: In your experience, are there opportunities for older people to raise their voices and have a discussion on health and well-being in their communities? Do you feel that older people have a voice in the decisions that are being made in your community/country? Do you feel that their opinions matter? Can you give me some examples? What are the ways that older people do/should participate in decision about their community?

#16: How could governments and communities, including younger people and other family members, promote intergenerational solidarity and foster healthy ageing for current and future generations?

**8.0 Conclusion section**

I want to thank you so much for taking the time to talk with me today. We would be happy to share the results of this study if you are interested.

#17 Are you interested in receiving the conclusions from this study (circle one)	Yes	No

**END OF INTERVIEW GUIDE**
