# Supplementary material for: The UN Decade of healthy ageing in practice: A qualitative case study on perspectives of healthy ageing among older adults, healthcare professionals, and decision-makers in Norway
Source: BMC Health Serv Res. 2026 Aug 14;26:1144. doi: 10.1186/s12913-026-15363-8 (PMC13488574; doi:10.1186/s12913-026-15363-8)
Supplement: Supplementary file 3 [file 12913_2026_15363_MOESM3_ESM.docx]

# List of abbreviations

SDG	Sustainable Development Goals

UN	United Nations

WHO	World Health Organization
